# Propagation of uncertainty in experiment: structures of Ni (II) coordination complexes

**DOI:** 10.1107/S1600577518006549

**Published:** 2018-05-30

**Authors:** Martin J. Schalken, Christopher T. Chantler

**Affiliations:** a School of Physics, University of Melbourne, Australia

**Keywords:** XAFS, information content, uncertainty, nickel coordination complexes

## Abstract

Accurate experimental XAFS (X-ray absorption fine-structure) data including uncertainties are required during analysis for valid comparison of results and conclusions of hypothesis testing on structural determinations. Here an approach is developed to investigate data without standard interpolation of experimental data and with minimal loss of information content in the raw data. Nickel coordination complexes bis(i-n-propylsalicylaldiminato)nickel(II) (i-pr) and bis(*N*-n-propylsalicylaldiminato)nickel(II) (n-pr) are investigated. The additional physical insight afforded by the correct propagation of experimental uncertainty is used to determine newly refined structures for the innermost co-ordination shell. Two sets of data are investigated for each complex; one optimized for high point accuracy and one optimized for high point density. Clearly both are important and in this investigation the quality of the physical insight from each is directly provided by measured and propagated uncertainties to fairly represent the relevant accuracies. The results provide evidence for an approximate tetrahedral geometry for the i-pr Ni complex that is more symmetric than previously concluded, with our high point accuracy data yielding ligand lengths of 2.017 ± 0.006 Å and 2.022 ∓ 0.006 Å for Ni—N and Ni—O bonds, respectively, and an even more skewed square-planar (*i.e.* rhombohedral) arrangement for the n-pr complex with corresponding bond lengths of 2.133 ± 0.004 Å and 1.960 ∓ 0.003 Å. The ability to distinguish using hypothesis testing between the subtle differences in XAFS spectra arising from the approximate local tetrahedral and square-planar geometries of the complexes is also highlighted. The effect of standard interpolation on experimental XAFS spectra prior to fitting with theoretical model structures is investigated. While often performed as a necessary step for Fourier transformation into position space, this will nonetheless skew the fit away from actual data taken, and fails to preserve the information content within the data uncertainty. The artificial effects that interpolation imposes on χ_*r*_^2^ are demonstrated. Finally, a method for interpolation is introduced which locally preserves the χ_*r*_^2^ and thus information content, when a regular grid is required, *e.g* for further analysis in *r*-space.

## Abstract 2

An approach to investigate XAS data without standard interpolation of experimental data and with minimal loss of information content has been developed. The additional physical insight accorded by the correct propagation of experimental uncertainty has been used to determine newly refined structures for the innermost co-ordination shell of Ni(II) coordination complexes.

## 1. Introduction   

X-ray absorption fine structure is the series of oscillations observed in the absorption spectrum following an absorption edge due to interference of the photoelectron wavefunction back-scattering from nearby atoms. Despite containing detailed information on the arrangement of atoms in the local region around the central absorber, high-precision determination of physical characteristics relies on accurate fitting of theoretical models with the experimental data. To date, data collection has not defined uncertainties nor propagated them into hypothesis testing and structure determination, with the exception of the X-ray extended range technique (XERT) (Chantler, 2009 ▸). While fingerprinting of XANES (X-ray absorption near-edge structure) and, for example, pre-edge features can be assessed to useful levels without least-squares analysis or principal component analysis, distinguishing alternate structures, ligands and shells needs a reliable 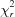 measure of goodness-of-fit (Koningsberger *et al.*, 2000 ▸; Chantler *et al.*, 2012 ▸).

Techniques including X-ray crystallography and neutron diffraction can be used in structure determination (Ladd & Palmer, 1977 ▸) to a high degree of accuracy, though they have obvious limitations when the sample of interest is in non-crystalline, solution or dilute form. The extended X-ray absorption fine-structure (EXAFS) region of the absorption spectrum is useful in determining the structure of isolated molecules (Sayers *et al.*, 1971 ▸; Eisenberger & Kincaid, 1978 ▸), and has proven to be a powerful tool in subtle structural determination (Mazzara *et al.*, 2000 ▸; Chantler *et al.*, 2012 ▸).

Following one of many standard pre-edge removals, the experimental data are transformed into 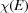 by
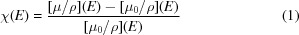
where 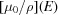 estimates the isolated atom background curve. Depending upon formalism, this pre-processing removes background effects from a matrix and solvent and any absorption or scattering from atoms not involved in the edge; defines and subtracts the edge energy or edge offset 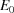, removes the edge-jump to the above-edge region, and estimates somehow an isolated-atomic absorption function above the edge. It is often incorporated into a single routine which makes an empirical spline fit through the data points above the absorption edge. This enables the isolation of the XAFS oscillations to allow structural determination. The spectrum is then converted to a function of wavenumber *k*,
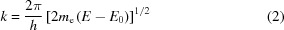
where 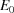 is the ‘edge energy’.

Much activity around the 1990s emphasized the need to fit spectra to allow structural insight, though the measures used varied quite widely and with non-uniform results. O’Day *et al.* (1994 ▸) introduced a goodness-of-fit measure but did not incorporate uncertainties or the standard deviation of experimental data. They stated that ‘there is currently no accepted method for determining these errors’. Similarly, Filipponi & Di Cicco (1995 ▸) comment that ‘any XAFS report should be accompanied by a detailed analysis of the statistical errors due to random noise in the raw spectra’. However, ‘general procedures to estimate errors … are still not well established’.

There have been attempts to estimate uncertainty for XAFS data. Dent *et al.* (1992 ▸) used a piecewise polynomial to extract residual noise hopefully free of any structure, and equivalently to use a Fourier filtering to remove dominant structure to hopefully yield a noise spectrum. Of course these are recursive methods and depend upon an ideal fit of any structure using empirical means in order to derive the variance and noise that would allow the structure to be determined.

Filipponi (1995 ▸) commented that the uncertainties on the fitted XAFS parameters should be given by the spread of such parameters resulting from variance from an ensemble of experimental spectra. However, he comments ‘unfortunately, only a single measurement is usually available’. He then provides three prescriptions for evaluating the noise distribution based upon an assumption of normal distribution of errors with assumptions of magnitudes of these multivariate distributions.

He suggests that a Metropolis Monte Carlo algorithm may be used to sample the parameter probability distribution. When applied to experimental data this will result in a sequence of independent sets of parameter values, each of which producing best fits of the experimental spectrum. The spread then represents the statistical uncertainty. This again is a post-facto representation and depends upon the initial determination of uncertainty. Finally, statistical errors can also be estimated from an assumption of perfect structural determination followed by a noise analysis of the residual, a little like that of Dent *et al.* (1992 ▸).

The *GNXAS* software estimates the noise in energy space: after fitting the XAFS structure, an error bar for each data point is generated by first fitting a polynomial of degree *q* < *M* over *M* data points, the residual square difference divided by *M* − *q* forms an estimate of the noise in the data. Repeating this along the spectrum allows for an uncertainty to be estimated at each point *via* interpolation (Westre *et al.*, 1995 ▸; Filipponi & Di Cicco, 1995 ▸; Filipponi, 1995 ▸).

An alternative approach is employed by the *IFEFFIT* software package (Newville, 2001 ▸), which estimates an un­certainty of experimental X-ray absorption spectroscopy (XAS) spectra as a function of wavenumber 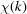 based upon a Fourier transform of *R*-space background, against theoretical models produced *via* the package *FEFF6* or recently *FEFF8L*. *IFEFFIT* is also the foundation for other software used in XAFS analysis, which often provide the benefit of a graphical user interface (GUI), such as the *ARTEMIS* and *ATHENA* packages (Ravel & Newville, 2005 ▸). The measure of model agreement in *IFEFFIT* 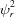 is calculated as 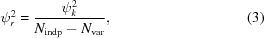


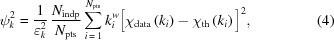
or alternatively 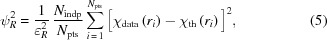
where 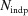 is an effective estimated ‘number of independent points’ in the XAFS spectra given by the Nyquist formula, 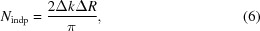
for a fit range of 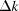 and 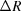 in *k*- and *R*-space, respectively (Stern, 1993 ▸).


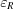
 estimates the uncertainty in the spectrum which is calculated as the root-mean-square of the Fourier-transformed data in a region at high *R*. Parseval’s theorem allows for conversion of this parameter into *k*-space with *w* the power of the *k*-weighted spectrum (Newville *et al.*, 1999 ▸),
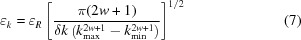
for data point *k*-spacing 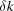. However, since most sources of noise are not taken into account, 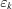 and 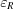 are underestimated, the error bars are too small, and 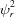 is overly large, and often 500–2000, compared with a more ideal propagated 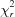.

In an attempt to remedy this, the fit is often re-evaluated using a somewhat arbitrary user-defined constant 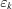 or 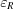 to yield a ‘good fit 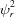 ≃ 1’ (Calvin, 2013 ▸). This assumes the final fit is perfect in order to define the uncertainties, and is therefore of limited use for hypothesis testing. The use of any such uniform error affects the fit since experimental uncertainties are non-uniform on 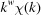
- or 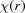-space. Without measuring the uncertainties experimentally, this skews the fit toward data points that actually have a large error, and away from those with small measured uncertainty.

Meanwhile Chantler’s high-accuracy analyses of QED and atomic spectra were extended into synchrotron research and X-ray absorption in several key papers. Commenting that estimates of statistical precision are critical (Chantler *et al.*, 1999 ▸), they made a series of (ten) considerations of key limitations of accuracy in X-ray absorption measurements to be addressed. This was followed by a detailed statistical analysis of noise and variance in synchrotron X-ray measurements and in ion chamber detection (Chantler *et al.*, 2000*a* ▸,*b* ▸). This explicitly measured numerous contributions to variance and precision, though indeed earlier authors had investigated some of these details for absorption. Finally this led to the first implementation of the XERT (Chantler *et al.*, 2001 ▸).

Essentially, to capture the actual uncertainty obtained in the experiment, one may begin with the variance of repeated measurements at each energy. Many experimental collection routines collect *n* multiple scans for the same sample of interest, where *n* may be from 3 to 10. XERT typically takes ten repeats for the same sample and aperture combination at each measured energy. XERT performs additional measurements to aid in the correction of experimental systematics such as dark current, so the calculation of final uncertainties in 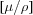 becomes less trivial, with procedures outlined by Tran *et al.* (2004 ▸), de Jonge *et al.* (2007 ▸), Chantler (2010 ▸) and Tantau *et al.* (2015 ▸). The determination of experimental uncertainties for 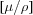 is further complicated in the case of a Hybrid experimental setup, especially for millimolar solutions, as is true for the data for this work (Chantler *et al.*, 2015 ▸; Islam *et al.*, 2016 ▸). When these uncertainties have been propagated to give uncertainty in 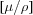, the results can be deposited or collected ready for data deposition, and it is best that this be done as part of the manuscript (Chantler *et al.*, 2001 ▸, 2015 ▸; Tran *et al.*, 2003 ▸, 2005 ▸; de Jonge *et al.*, 2005 ▸, 2007 ▸; Glover *et al.*, 2008 ▸; Islam *et al.*, 2014 ▸, 2016 ▸; Tantau *et al.*, 2015 ▸).

In most cases, the absence of a derived uncertainty in 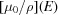 implies that such an uncertainty cannot be propagated to an uncertainty in 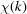 or 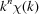. However, in recent work (Islam *et al.*, 2015 ▸, 2016 ▸) the uncertainty has been propagated to 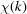 or 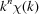 explicitly from the standard error uncertainty 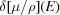,
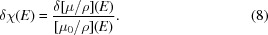
However, the data are then *always* interpolated onto a regularly spaced grid in *k*. This distorts experimental values, point density, information content and experimental uncertainties (Islam *et al.*, 2014 ▸). The change of point spacing will skew the fit toward a different region of the spectrum, hence any additional time spent during the experiment in particular energy regions to gain high detail, high point density or high point accuracy on features of interest will effectively be lost. These issues are general in XAFS and apply to common packages including *IFEFFIT*, *ARTEMIS*, *GNXAS* and *FDMX* for example.

**Figure 1:**
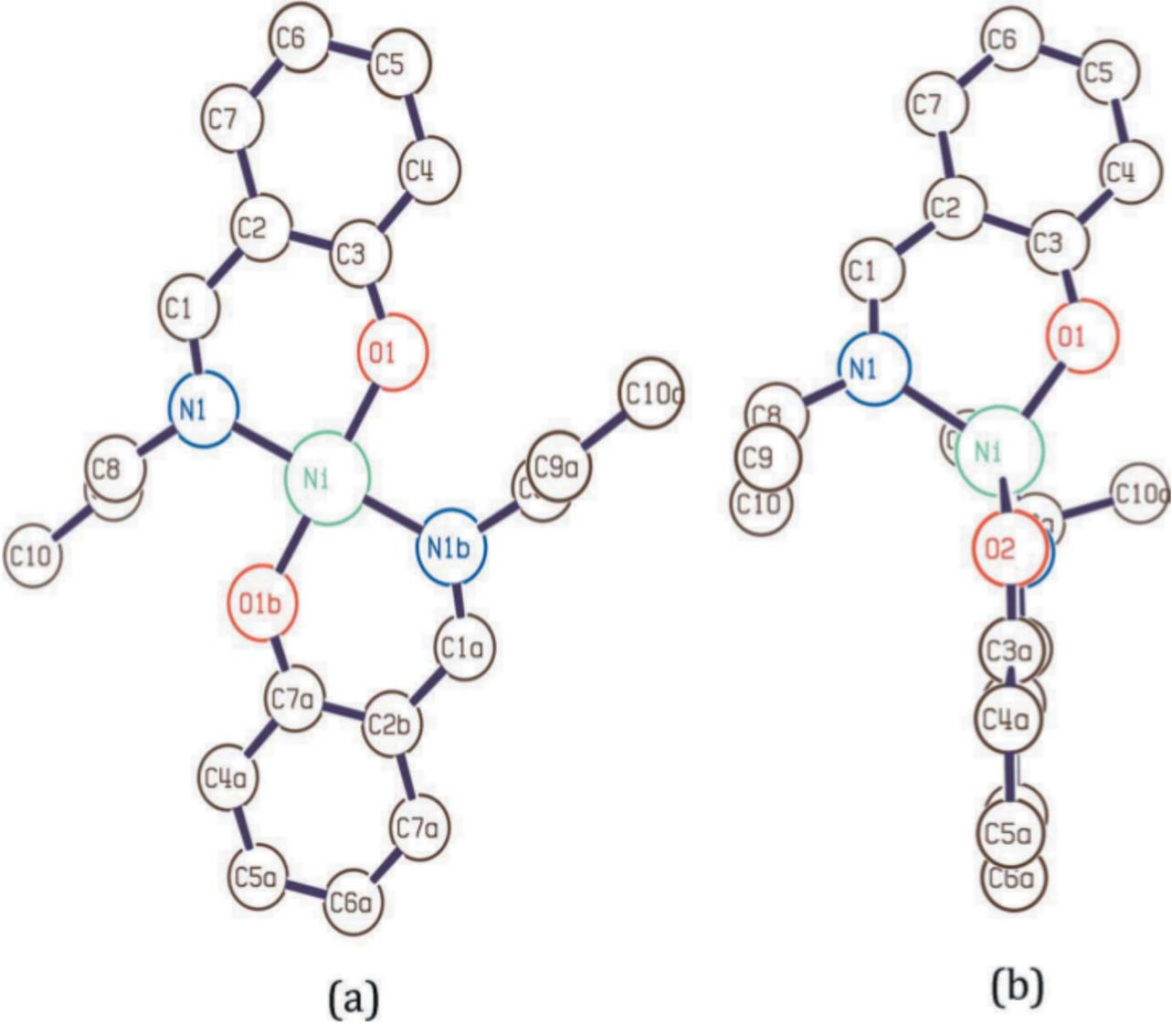
Molecular structures of the n-pr (*a*) and i-pr (*b*) Ni coordination complexes showing local tetrahedral and square-planar geometries around a central Ni atom (omitting hydrogen atoms).

In this work we extend the work of Islam *et al.* (2015 ▸) by examining the XAS spectra taken of Ni (II) complexes (Chantler *et al.*, 2015 ▸). The nickel complexes bis(i-n-propylsalicylaldiminato) nickel(II) (i-pr), and bis(*N*-n-propylsalicyl­aldiminato) nickel(II) (n-pr) (Fig. 1 ▸) are known to have local metal environments of approximate tetrahedral and square-planar geometries, respectively (Fox *et al.*, 1964 ▸; Britton & Pignolet, 1989 ▸). X-ray crystallography has been used to examine the solid state structures, and, while small variations in the inter-atomic distances are observed, it is confirmed that the overall molecular geometry of the solid state structures are maintained in solution. Hence these Ni complexes provide a convenient platform on which to demonstrate the sensitivity of XAFS to such differentiating characteristics. Besides being the subject of many structural and stereochemical analyses, salicylaldiminato Ni(II) complexes are also used as a catalyst in the polymerization of olefins (Chan *et al.*, 2000 ▸; Lu *et al.*, 2011 ▸).

Normal XAFS is considered to be able to distinguish the coordination number to 25%, whereas in this case the difference is 0%. Further, the bond lengths and path differences are essentially identical, making any comparison of structure or distinction of one hypothesis (square planar) *versus* another (tetrahedral) an extremely fraught problem. It is therefore an ideal example to compare the importance of uncertainty propagation and its consequence upon structural determination.

We will perform fits on non-interpolated spectra to evaluate the information content and to provide more reliable parameter and structure determination for these challenging coordination complexes. We will therefore:

(i) Develop a novel method and code for transforming the raw data while maintaining point density and point accuracy, with code in the supporting information.

(ii) Determine newly refined local structures based on non-interpolated experimental data for both high point accuracy data sets and high point density data sets.

(iii) Highlight the ability of XAFS as a powerful tool in stereochemical analysis.

(iv) Demonstrate the effects standard interpolation has on 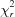 and the consequences on the interpreted structure.

(v) Present a new method of interpolation which preserves information content.

(vi) Explain that the new approach is not only effective across the whole range of the spectrum but also with respect to the distribution of data point density and accuracy across the spectrum.

## 2. Data for this analysis   

A total of four experimental data sets are used herein, gathered *via* the Hybrid technique, which simultaneously records XAS fluorescence and transmission data (Chantler *et al.*, 2015 ▸). The Ni complexes are in a frozen solution of 15 m*M*, kept at a temperature of ∼80 K, so as to observe and quantify structure in the absence of thermal disorder. For each complex, an XAS transmission spectrum is collected following high-accuracy Hybrid methodologies (Chantler, 2010 ▸), providing experimental data with high point accuracy (HPA) and with the spectra corrected for experimental systematics including energy calibration, dark current, harmonic contamination and scattering using published methods (Tran *et al.*, 2004 ▸; Glover & Chantler, 2009 ▸; Barnea *et al.*, 2011 ▸; Islam *et al.*, 2014 ▸; Tantau *et al.*, 2015 ▸). Additionally, a spectrum is also taken using a faster method for each complex, which trades accuracy for higher point density, in an experiment where time constraints did not permit both. Tabulations of the collected spectra can be found in the supporting information of Chantler *et al.* (2015 ▸).

## 3. Fitting data with original point density and point accuracy: *mu2chi*   

**Figure 2:**
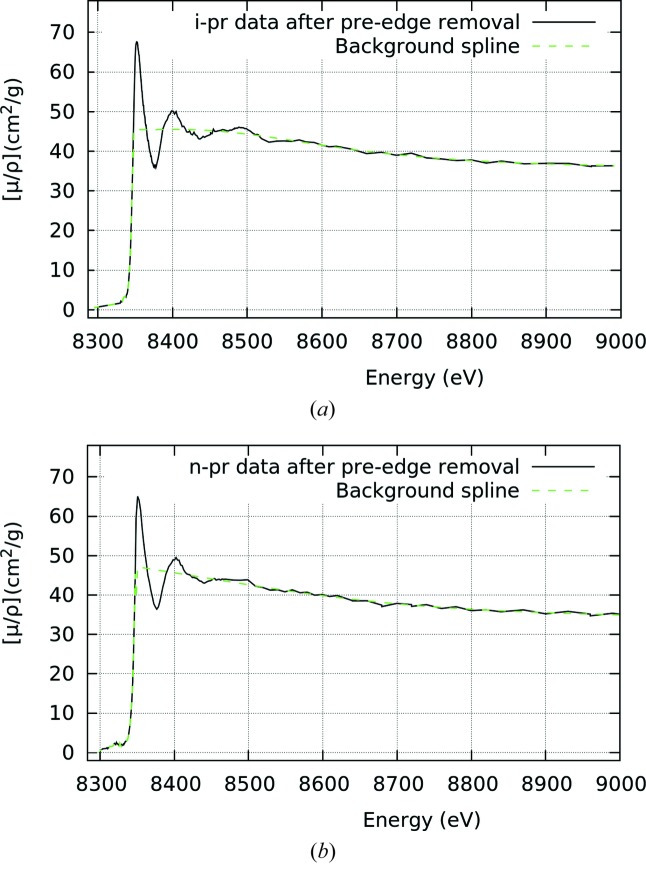
Quality of the data and background spline for the solvent matrix-removed absorption spectrum for i-pr (top) and n-pr (bottom) for the high point accuracy (HPA) data set.

**Figure 3:**
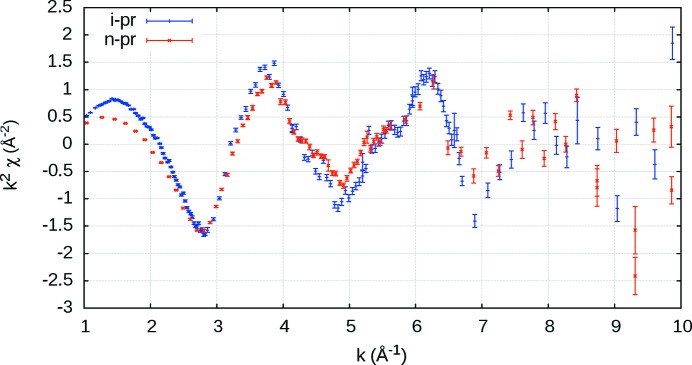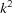-weighted *mu2chi* (non-interpolated) output for i-pr and n-pr from the HPA (Hybrid) experiment.

In order to use, preserve and propagate the information content contained within the experimental uncertainties, we introduce the *mu2chi* processing to convert XAS spectra from 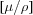 absorption *versus* energy *E* into χ *versus* *k* (*i.e.* it translates *mu* to *chi*) which avoids any interpolation and propagates experimental measurement uncertainty. Fig. 2 ▸ shows one of the stages of the *mu2chi* data reduction, whereby the background spline is removed. An example of a final *mu2chi* output is shown in Fig. 3 ▸. We provide the (general) code, manual and makefile in the supporting information.

There exist numerous methods for interpolation. Previous work (Islam *et al.*, 2015 ▸) utilized a cubic spline approach. After the pre-edge and background (Fig. 2 ▸) are subtracted in the conventional manner, a cubic fit with standard deviation uncertainties is made through four data points, and is evaluated at a regular 0.05 Å^−1^ spaced grid, iteratively stepping through the data. Each point on the grid then has multiple fitted points with uncertainties, with the final value determined by using a weighted mean, and the uncertainty being a weighted standard deviation.

**Figure 4:**
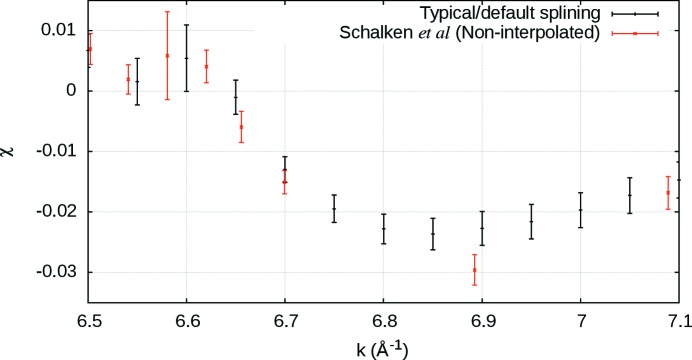
A typical splining method (Islam *et al.*, 2015 ▸) (black) compared with the new non-interpolated method (red). Smoothing is apparent around 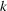 = 6.9. Even when interpolated and non-interpolated approaches share a common *k*-value, the data point differs, *e.g.* at 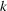 = 6.7. The new non-interpolating approach does not distort data, structure or uncertainty.

This is common, but often the interpolated value will differ from that of the original, despite being located at the same *E* or *k* value (Fig. 4 ▸). This then does not reflect the real data, and measured outliers are often omitted by the spline, incorrectly improving the reported 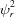 or 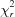.

## 4. *eFEFFIT*   

The theoretical spectra are calculated following the photoelectron wave model (Lee & Pendry, 1975 ▸; Barton & Shirley, 1985 ▸), and expressed as a sum of photoelectron scattering paths through the XAFS equation (Zabinsky *et al.*, 1995 ▸; Bunker, 2010 ▸),
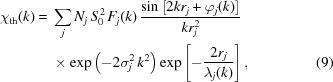
where 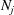 is the degeneracy of the path, 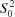 corresponds to many-body reduction effects, approximated as constant, 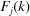 is the backscattering amplitude function, 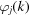 is the phase shift, 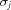 is the Debye–Waller factor for thermal movements, 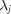 is the photoelectron mean free path, and 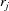 = 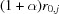 is the half path length, with α being the relative scaling due to thermal expansion.

We introduce a modified version of the *IFEFFIT* subroutine *FEFFIT*, called *eFEFFIT* (*error-FEFFIT* or *error-FEFF fit*), which allows for experimental uncertainties to be input and propagated. This should be used when determining the fit, as well as in the determination of 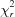 [equations (10) and (11)], so as to better reflect the actual data and their significance (Smale *et al.*, 2006 ▸), 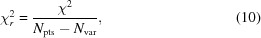


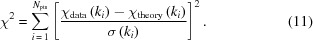
The numerator of equation (11) is the residual and is calculated as the difference between 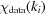, the *i*th experimental data point, converted to a function of wavenumber *k*, and the corresponding theoretical modelled value at that point 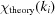. 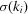 is the associated propagated uncertainty. 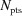 represents the number of points inside the fitting range, and 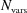 the number of fitted parameters. The authors plan to distribute *eFEFFIT* code in the near future.

We recommend to not interpolate the experimental data onto a regular grid but rather interpolate the theoretical model onto the experimental data point array. Otherwise, it is difficult to preserve the information content of the original experiment during interpolation. Experimental data points should of course be taken in at least semi-regular points in *k*-space; however, this is dependent upon an exact and correct determination of 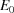 prior to data collection, which is generally implausible. Also, there can be a focus on local structure or multiple edges which makes a more uniform scan impractical. If the experimental point density varies greatly across the fitting range, the fit will be dominated by regions of high point density.

## 5. *eFEFFIT*, *IFEFFIT* fitting   

Our starting point for structural refinements in this work will be the structure presented in Table 4 of Islam *et al.* (2015 ▸). This represents an intermediate stage of analysis whereby lengths are refined yet the N—Ni—O angle is fixed at 90°. The model includes the carbon rings but omits the H atoms (Fig. 1 ▸).

Fitting should be done in *k*-space as opposed to *r*-space, otherwise interpolation to a uniform grid is still required for fast Fourier transform, compromising further the information contained in the measured data points. We perform the fit without any *k*-weighting to avoid emphasizing different regions of the spectrum. Graphs throughout this work, showing a *k*-weighted spectrum, are scaled after the fit.

The theoretical models used are provided *via* *FEFF8*, with parameters RPATH = 4.85, being the maximum half scattering-path length, with the maximum number of legs NLEG = 6 being applied consistently throughout both this and previous (Islam *et al.*, 2015 ▸) work. Table 1 ▸ shows the fitted parameters using the previously refined model, and conventionally spline-interpolated data compared with the newly processed χ *versus* *k* non-interpolated experimental data. The uncertainties look similar but the fit might appear worse as 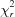 is around double for the ‘correct’ raw data rather than the interpolated form. The interpolation smooths the data and ergo reduces apparent noise; but it does so artificially and hence distorts the spectrum and the apparent fit. Notice that there are small differences in the scale of thermal parameters but that all are physical and the nearest neighbours have a smaller thermal broadening.

The results obtained previously (Islam *et al.*, 2015 ▸), and in Table 1 ▸ for comparison, only implemented experimental uncertainties σ in the post-fit calculation of equation (11). Henceforth we implement the *eFEFFIT* routine to utilize the experimental uncertainties in both determining the fit and in the calculation of 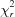. Table 2 ▸ shows the effect of utilizing experimental uncertainties in the fit in addition to using the raw (non-interpolated) data. This maintains the understanding of significance testing with experimental uncertainties, and entirely eliminates any *ad hoc* estimation of uncertainties. The analysis can then test the validity of theory, model, experimental uncertainty and structure. In Table 2 ▸, most parameters only shift a small amount, so the use of raw data and uncertainties might be seen as not so important; however, the quoted uncertainties are generally halved, some shifts are equal to one derived standard error, and the major changes will be seen in Tables 3 ▸ and 4 ▸.

Using the full *eFEFFIT* and non-interpolated data, a third step is now to perform a refinement of the key Ni—N and Ni—O bond lengths and N—Ni—O inter-atomic angle. Since the approximate geometries of each complex are known, the refinements for the i-pr and n-pr complex will be based on the tetrahedral (TD) and square-planar (SQ) theories, respectively.

**Figure 5:**
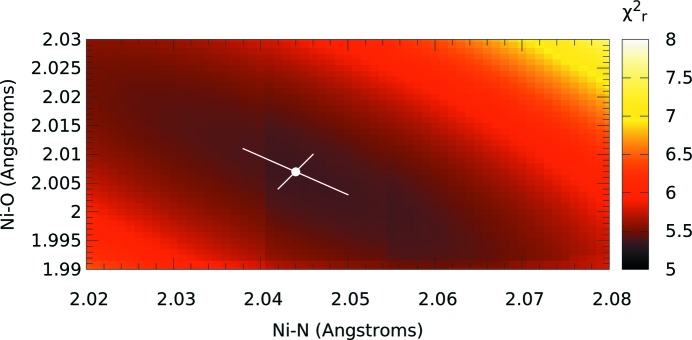
Refinement of i-pr bond lengths by minimizing 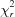 in two-dimensional parameter space, while the angle is held at 90°. The white spot marks the location of the minimum, 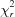 = 5.35. The white lines show uncertainties along perpendicular axes aligned with the valley, with the magnitudes corresponding to a percentage increase in 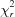 equal to that of the percentage uncertainty of the fitted α parameter (0.29%). Note the asymmetry of the valley (see text).

The first step is a two-dimensional refinement performed on the key bond lengths, keeping the O—Ni—N angle at 90°, followed by a one-dimensional angular refinement scan using the new bond lengths. The new fitted parameters are presented in Table 3 ▸. Only the nitrogen and oxygen atomic positions are being modified while carbon coordinates remain fixed. The 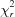 surface is presented in Fig. 5 ▸ and indicates the difficulty of the minimization to clearly distinguish the N from the O, as expected. The neighbouring carbon atoms and those in the rest of the molecule also undergo small changes in their positions to compensate for the new nitrogen and oxygen locations.

The 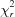 valley is quite shallow (Figs. 5 ▸ and 6 ▸). The asymmetry of the valley in Fig. 5 ▸ indicates a relatively firm positioning of the distance of the nitrogen and oxygen atoms from the nickel centre, while allowing more flexibility as to the interchanging of their relative proximity. Also indicated is a sharper definition of the oxygen location than the nitrogen. A key result of this refinement stage is a more plausible 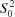. Another significant consequence is that the bond radii have shifted significantly from the result of the previous fit using spline-interpolated data without uncertainties. Hence the importance of the raw data with uncertainty for any quantitative analysis.

**Figure 6:**
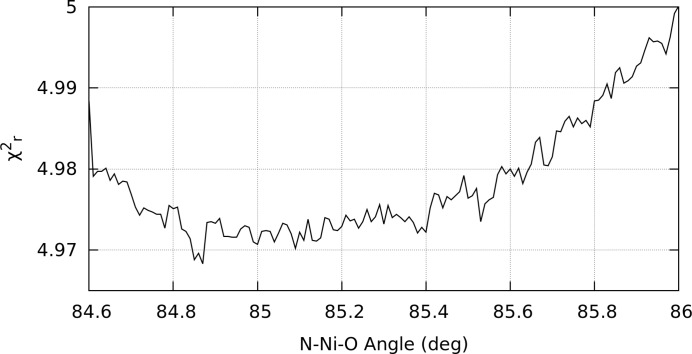
One-dimensional angle scan to locate minimum 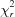 for the i-pr complex, with bond lengths from Fig. 5 ▸. The minimum 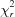 is estimated at 85°.

All refinements exhibit small discontinuities in 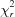 as a function of bond and angle (Fig. 6 ▸). These occur primarily from two sources. Changes in key inter-atomic distances result in the relative contribution of certain paths crossing the threshold for acceptance criteria as set in *FEFF*. Re-defining this parameter simply results in the discontinuities occurring elsewhere, and its omission produces too many unique *FEFF* paths for the software *IFEFFIT* to handle. Secondly, altering the bond lengths causes some paths to pass into or out of the maximum path length, also defined in *FEFF*. An effort to circumvent this artifact was to use only a few three-legged paths of any length, although a change in the position of the minima occurs. However, the overall gradient trend exists over subsections of the scan partitioned by the discontinuities. Hence the location of the minimum is largely unaffected by these small discontinuities.

The uncertainties of the refined bond lengths correspond to the fitted percentage uncertainty given for the α parameter. Uncertainties of the N—Ni—O bond angle are determined by matching the percentage increase in 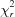 to that for the bond length uncertainties. This latter method applied to bond lengths (Fig. 5 ▸) is in good agreement with the former more direct method of uncertainty determination. This reflects and is consistent with the expectation value for the standard error uncertainty corresponding to an increase of 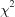 of unity.

There is no guarantee that the lowest value of 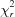 in three-dimensional parameter space has the bond length values determined in the two-dimensional length search. Therefore we now perform a simultaneous three-dimensional refinement with the newly refined bond lengths and angles (Table 4 ▸). Indeed we find that the minimum from such a rigorous search is significantly different from that from a two-dimensional and one-dimensional search. The earlier restricted search is common but is not sufficient in a flat valley. Hence restricted minimizations, while necessary, are causes for concern in quantitative analysis. Of course we all have molecules where the full number of independent degrees of freedom cannot be fitted as there is insufficient information content in the spectra. The solution is to constrain the physical independent motions to the most significant with chemically meaningful constraints, and to preserve the maximum information content of the data. If needed of course, collect better data.

Having found suggested structures for each of the complexes, we should wonder whether the structures are simply local minima. Indeed, is the ‘square-planar’ molecule optimized with the data set to be square planar, and is the ‘tetrahedral’ molecule optimized to be tetrahedral? Conversely, might they be indistinguishable by XAFS analysis because the paths, especially two-legged paths, are basically identical?

**Figure 7:**
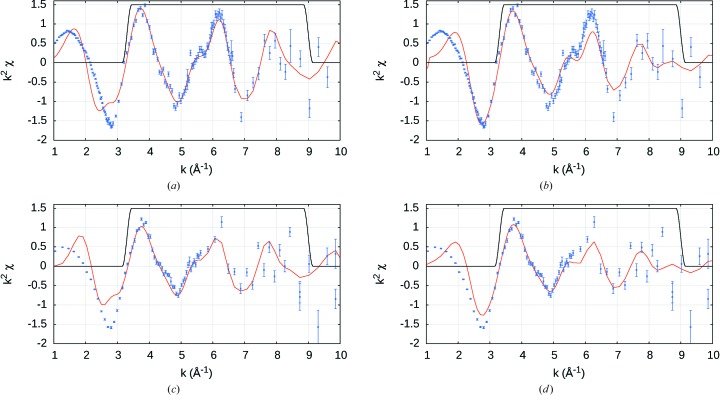
Cross-fits of 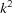-weighted XAFS spectra using Hybrid (HPA) non-interpolated data with refined structures from Table 4 ▸. Top row: i-pr experimental data; bottom row: n-pr. Left column: fitted with the optimized tetrahedral structural model; right column: fitted with the optimized square-planar model. The black line is a Hanning window and indicates the region over which the fit is performed. Inferior fits are obtained when alternate *FEFF* structure geometry is applied in (*b*) and (*c*), as detailed in Table 4 ▸.

In other words we need more serious hypothesis testing, by attempting to fit the ‘square-planar’ molecule with the tetrahedral structure; and to fit the ‘tetrahedral’ molecule using the square-planar structure. Can we distinguish the two conformers by XAFS analysis? Hence after refining the structure with the appropriate geometry, each complex is then fitted with the alternate model. The resulting cross-fits are inferior, confirming the correct stereochemistry for each moiety (Table 4 ▸, Fig. 7 ▸).

The uncertainties presented throughout this manuscript reflect the standard error (s.e.) for the parameter. This is exactly the same meaning for any error analysis on the assumption that all variates are normal, Gaussian-distributed and independent. Uncertainties were determined in a manner consistent for all parameters and the same as the basic methodology of *IFEFFIT*. The uncertainties reported are fully consistent with a detailed mapping of 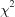 and 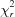. In other words, queries should be made, where there are occurrences of discrepancies between analyses of much more than 3 s.e.

Table 4 ▸ shows better defined, more robust, bond lengths when a three-dimensional parameter search is performed, especially for the n-pr complex. Significant shifts in bond length and angle are observed compared with the earlier tests, with the i-pr complex gaining a higher level of symmetry. The cross-fits (i-pr/SQ and n-pr/TD) yield larger values of 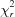 indicating that the subtle changes in geometry are correctly modelled by the fits from the data, and that the hypothesis testing is able to distinguish the two conformations. The thermal parameter 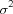 is significantly larger and less well defined for the square-planar structure than for the tetrahedral structure.

Table 5 ▸ re-presents results from Islam *et al.* (2015 ▸), showing *IFEFFIT*-fitted values for cross-fitting of the theories. As before, TD and SQ model structures are based on i-pr and n-pr data, respectively.

Ni—N and Ni—O bond lengths in the previous literature were quite symmetric in both the tetrahedral and square-planar theories, as opposed to our results propagating experimental uncertainties without interpolation (Table 4 ▸). Similar trends in the fit parameters are seen. However, the inter-atomic angle shows a noticeable difference between the theories in our results, which is not as evident in Table 5 ▸.

This disagreement with the results in Table 4 ▸ shows the significant difference of structure and ergo error resulting from the use of spline interpolation without using fitting uncertainties, and illustrates by contrast the considerable changes that occur when the data and uncertainties are processed with a rigorous error-preserving method. It also suggests that using the non-interpolated data to fit the theoretical model allows for a sharper differentiation between the geometries of the individual complexes, and between hypotheses of structure or dynamics in general.

## 6. High point density (HPD) XAFS analysis   

The discussion above used spectra collected to emphasize high point accuracy so that each point carried insight as to the physical structure of the complexes. With this Hybrid method, independent measurements are made on a range of possible systematic contributions to the signal and each data point should have a small uncertainty. The HPA spectra have some large spacings in regions of *k*-space which may hide critical information. Relative changes of XAFS from point-to-point carry the most important information about structural changes and so a sufficiently fine point spacing to reveal and represent the frequencies corresponding to particular paths and bond distances is important. The HPD spectra are intended to cover all important structural frequencies with uniform stepping in *k*-space, but with lower point-wise accuracy, very like the standard continuous scans or the standard QuickXAFS scans. The HPD data are stepped but in approximately equal steps in *k*-space, and with some increased counting for higher *k* to better match statistics. It is interesting and useful to see how these choices affect the structural determination and how the non-interpolation is affected with different spacings and uncertainties. Therefore we repeat the last few steps in applying the above logic to the corresponding HPD spectra. This may also ask if the structural conclusions from the HPA spectra are confirmed or otherwise by appropriate analysis of HPD spectra, or by other different measurement cycles for the same sample and structure.

A simultaneous refinement of the key inter-atomic bond distances along with the N—Ni—O angle is therefore carried out by performing a grid search over all three parameters and fitting the *k*-range at each point.

**Figure 8:**
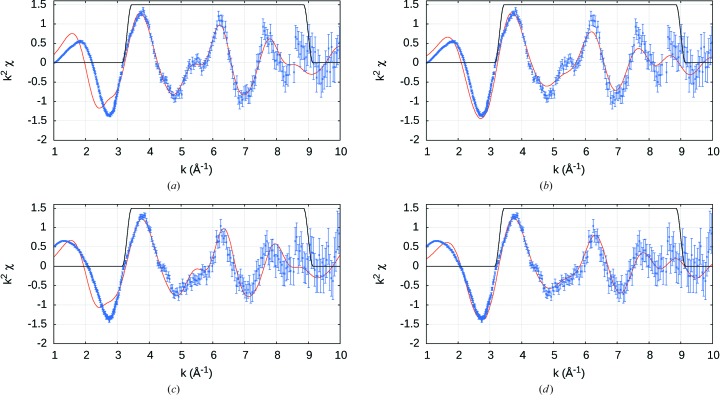
Cross-fits of 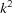-weighted XAFS spectra using Hybrid (HPD) non-interpolated data with refined structures from Table 6 ▸. Analogous to Fig. 7 ▸.

The fits with the theoretical models are depicted in Fig. 8 ▸. The SQ column of Tables 4 ▸ and 6 ▸ contains set values for certain *IFEFFIT* XAFS-fitted parameters, namely 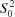 and 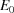. This was done to prevent the software from fitting unphysical values, which would also have the additional negative effect of invalidating any cross comparisons. Ideally all parameters might be free; but their correlation and the limited information content prevents this. Hence they should be modelled with chemically and physically plausible restraints and constraints to yield a maximal search through parameter space of the most significant structural parameters. The number of fitted parameters 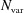 [equation (10)] changes by one due to the fixing of a parameter, and only changes 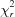 by 1–2%. Hence no conclusions are affected by the small change of 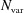.

Generally, there is good agreement between the results from the HPA and HPD data sets: the SQ/n-pr fit yields a higher degree of asymmetry in Ni—N and Ni—O bond lengths than the previous literature (Table 5 ▸). The TD/i-pr structure also exhibits properties similar to that of the HPA results, with similar Ni—N and Ni—O distances. However, these were found here to be inverted in the HPD data, with the nitrogen closer than the oxygen. The amplitude reduction factor, 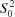, in the SQ/i-pr fit has a slightly large value. The structure found for the square-planar model possesses slightly larger bond lengths than for the HPA data set, with a relatively higher uncertainty on the alpha scaling parameter. Cross-fitting the experimental data with the opposite model structures again produces inferior fits and again demonstrates the ability of error propagation without interpolation to distinguish important hypothesis testing including of subtle geometric changes.

Given the stated error on the bond lengths shown in Tables 4 ▸ and 6 ▸, it is reasonable to question whether the corresponding resolution in *r*-space is sufficient to separate the peaks from the N and O atoms. However, it must be realized that one only needs to observe a shoulder in the peak of the radial distribution. Such asymmetry implies multiple radii, and can hence be expressed as independent locations to within the given uncertainty. Given a FWHM resolution of approximately 0.2–0.3 Å it is realistic to identify individual peaks of similar magnitude with separations of 0.18, 0.17 and 0.07 Å. We do note that the HPA data for the TD geometry have a separation of only 0.05, and hence do not conclude which one is shorter, and this particular data set is consistent with the distances being identical.

It is also observed that the HPA data were not able to distinguish a difference in key bond lengths for the tetrahedral arrangement, whilst the HPD did. This is simply explained by the fact that one of the data sets contained more incisive information relating to this specific question, since the interference waves from each were better defined in the HPD. This shows that one particular method, HPA or HPD, is not ‘better’ than the other, as it depends on what information is desired. Ideally, one should have the high point density, with each point having the accuracy obtained from the HPA method.

The final Table 4 ▸ (HPA) and Table 6 ▸ (HPD), while different data sets, should be the same structure and consistent within uncertainty: if both are evaluated to accurate levels; if the input data uncertainties are estimated accurately; if the model is a true representation of the structure observed (both the theory and the imputed structure); and if the respective data sets reflect the same structure *etc*. In most cases this consistency is clear from the tables, though the uncertainties on any given parameter vary as they should. One must also consider the level of agreement between the structures resulting from the HPA and HPD data sets for the i-pr sample. The HPA i-pr yielded key bond lengths of 2.017 Å and 2.022 Å for the N and O distances, respectively, with a stated uncertainty of 0.005 Å. In general, 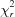 should be unity *if* the model is correct (*i.e.* perfect theory and exact structure). If not unity, then it is possible that the input data uncertainties are incorrect and underestimated by 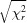, and hence *if* the model is exactly correct then the resulting uncertainties can commonly be reported as (standard error) × 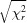. Assuming the uncertainties are complete as stated, the shifts from Table 4 ▸ to Table 6 ▸ for the N and O distances are 2.017 − 1.985 = 0.032 Å and 2.055 − 2.022 = 0.032 Å or some 4.1 s.e.; or 2.2 including 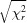. Hence the results are plausible and consistent, but do reveal shifts and sensitivities from different data sets.

## 7. Interpolation of experimental data with minimal distortion of information content   

We now address the question of how best to preserve the information content of a data set if it *must* be interpolated onto a linear grid, in this case in *k*-space. We have argued that in general such a grid is liable to distort features and weight spectral ranges inappropriately, and also to scale noise in the wrong way. How then can we envisage an attempt at interpolation onto a regular linear grid in, for example, *k*-space which will approximately preserve the information content overall and especially of each region of the fitting range? Knowing and having demonstrated the errors of the popular spline interpolation used in the majority of packages, we first look at the popular and common cubic interpolation, followed by an approach to preserve the information content such that the 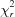 produced remains constant to within some small margin.

For a cubic interpolation, the value of 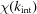 can be determined by fitting a cubic function between the points 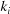 and 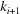, where 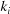 < 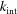 < 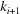. The points at 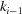,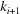 and 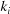,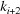 are used to estimate the gradient at points 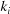 and 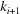, respectively. Then these along with the values of 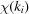 and 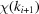 are used to determine the four coefficients in the polynomial equation 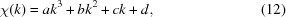
so that 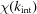 may be calculated. A simple linear interpolation is performed on the first and last data intervals. In this manner we preserve all points of the data if the *k*-grid point is at the same point as the data, and we preserve the local average derivative to yield a smooth curve of interpolation that passes through all the data. This method is also common and useful in preserving a smooth first derivative in the interpolation. In other words, we do *not* use the standard spline which smooths and replaces data points. In this cubic interpolation, we can define interpolated uncertainties as
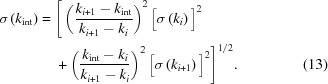
This interpolated uncertainty yields exactly the original uncertainty at the original data points, so in that sense this is information-preserving. Also, this prescription has one advantage that the predicted uncertainty is the corresponding uncertainty for a single interpolated point.

In a given interval between raw data points, interpolation of data will either introduce extra points or fail to place a point at all. Whenever an interval has no interpolated point, there will necessarily be loss of information content. Therefore, there is an implicit expectation or requirement that a sensible interpolation approach should always have at least one point in any range of the original experimental spectrum; that is, that all interpolation approaches ought to have a grid spacing 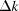 less than or equal to the smallest grid spacing 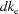 in the experimental data set across the range of interest, or across the range to be fitted.

Unless proper care is taken, the changing number of points in the summation in equation (11) influences 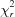. Even if 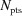 were to be maintained, the implementation of a regular grid over non-uniform experimental data collection will bias the fit toward different regions of the spectrum. Table 7 ▸ shows the significant impact which interpolating the experimental data typically has on the resulting 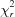.

Simply put, this interpolation routine should (always) introduce additional points into the data set, but if for example the two end points of a grid remain in the interpolation then any additional point introduced with any uncertainty will appear to add to the information content of the data incorrectly; that is, if the end-points correctly represent all the information content of the data, then any additional interpolated point requires a weighting of zero to retain information content.

## 8. Information preservation   

Whilst, obviously, there are many interpolation methods available, none of them (locally) preserve the information content contained within, and certainly nothing of the kind has been applied to XAFS. Current *IFEFFIT* and *ATHENA* software changes data values and data uncertainties, and hence are manifestly not information preserving. Our method which we present in this section preserves variance over a range of interpolation and parameter space, which is required for a non-distorted fit to be obtained.

Upon examination of the formula for 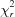 it is evident that in order to preserve 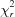 one must preserve the following quantity for each interpolation interval to remove region bias,
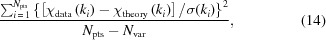
where the residual Res = 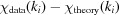.

We also define
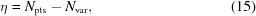
where 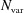 is the number of *IFEFFIT* fitted variables (in this case four, *i.e.* 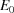, α, 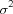 and 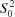) and 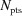 is the total number of data points in the range over which *IFEFFIT* will eventually perform the fit over. Therefore, this range should be entered into the *mu2chi* program and re-run should the desired fitting range change. 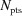 for each of our four data sets are shown in Tables 4 ▸ and 6 ▸. The residual is calculated post-fit, and the adjustment to the uncertainties is performed in the earlier *mu2chi* process. Therefore we will now make the assumption that the residual is constant over the interpolation interval, and bring it out of the summation. Hence for each interval, we now aim to satisfy
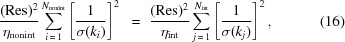
where *N*_nonint_ and *N*_int_ are the number of points present inside the given interpolation interval, for the non-interpolated and interpolated data, respectively. For the non-interpolated data set, the number of points in the interval is taken to be unity, with the contribution to the 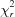 from the uncertainties of the interval endpoints divided equally to the two respective adjacent intervals. As the residual is assumed to be constant whether interpolated or not, these cancel. Thus one requires
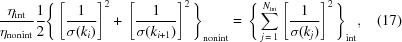
whence
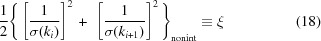
is the sum of the inverse of the uncertainty squared for the local non-interpolated interval.

**Figure 9:**
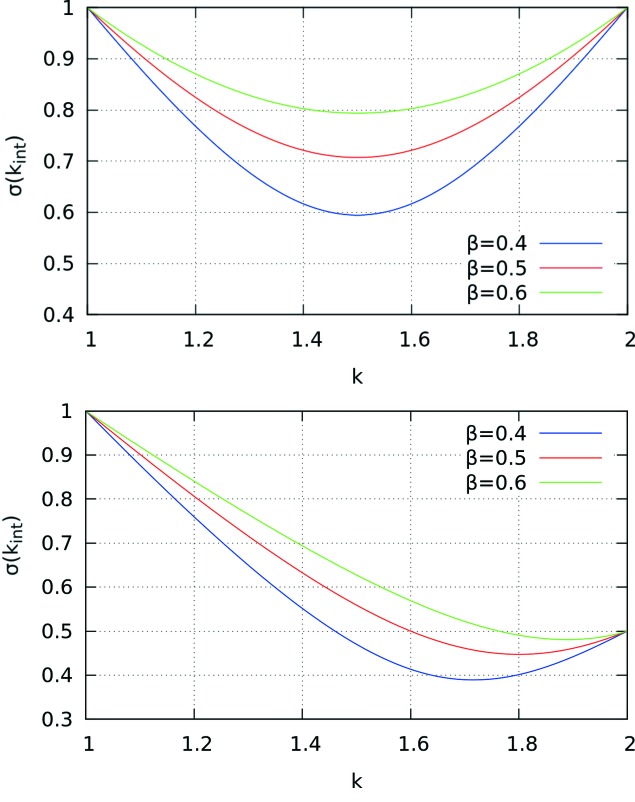
Curve tracing out error bar endpoints for a single interpolation interval with changing β parameters. Top: symmetric error bars of unit magnitude for endpoints of 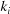 = 1 and 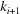 = 2. Bottom: asymmetric endpoint error bars, with 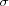 = 0.5 at 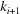 = 2. The curve with β = 0.5 shows the result of the standard cubic error bar interpolation [equation (13)].

In order for equation (17) to be true, we observe that the following expression must be unity,
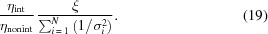
Therefore, following the error bar calculation as per the non-information-preserving approach of equation (13), this value is determined for each interpolation interval. Should this value not be unity, the uncertainties within the interpolation interval are adjusted by altering the functional ‘height’ of the curve joining the tops of the error bars of the interpolated points. For this purpose, we introduce a parameter β, the incrementing of which allows the fine adjustment of the value of the error bars in a manner depicted in Fig. 9 ▸. β is adjusted in steps of 0.001 to convergence, until either equation (19) is equal to unity within some predefined level of tolerance (0.01%), or a failsafe mechanism is triggered after 1000 iterations. This is performed using
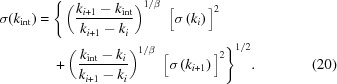
When β equals 0.5, this expression is reverted back to that in equation (13). The process is performed on each interpolation interval.

Table 8 ▸ shows how the information-preservation procedure of *mu2chi* works to restore 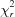 to similar levels to that from fitting with the non-interpolated data. The HPA data have been restored to within 10% of their respective original 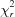. Conversely, the data with a denser data point spacing, HPD, now show a 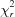 significantly less than the non-interpolated version, which is too low and does not reflect the original experimental data. This large discrepancy for the corrected HPD data is explained by considering the spacing of the interpolated grid, which in Table 8 ▸ was 0.05 Å^−1^. At this spacing, many pairs of adjacent non-interpolated data points contain no interpolated data point between them, and so a contribution to the summation in equation (11) is lost, falsely lowering the reported 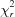. While the cause might be considered obvious, ensuring that this does not occur for your data is important.

The HPD data were taken *via* a single monotonic energy sweep, with data acquired at regular *k*-spacing based on an estimate of 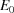. This then requires a small amount of interpolation, for which our method is perfectly applicable, with the number of interpolated points approximately equal to the number of points in the raw data. The β value depends on the fractional distance from the endpoints. The monotonic energy sweep in *k*-space is very similar to the continuous energy scans often used, which may be uniform in angular velocity, energy spacing or in principle *k*.

The following section examines the effect changing the interpolation spacing has on the resulting 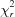 for all four data sets.

## 9. Selection of *k*-spacing of the interpolation grid   

**Figure 10:**
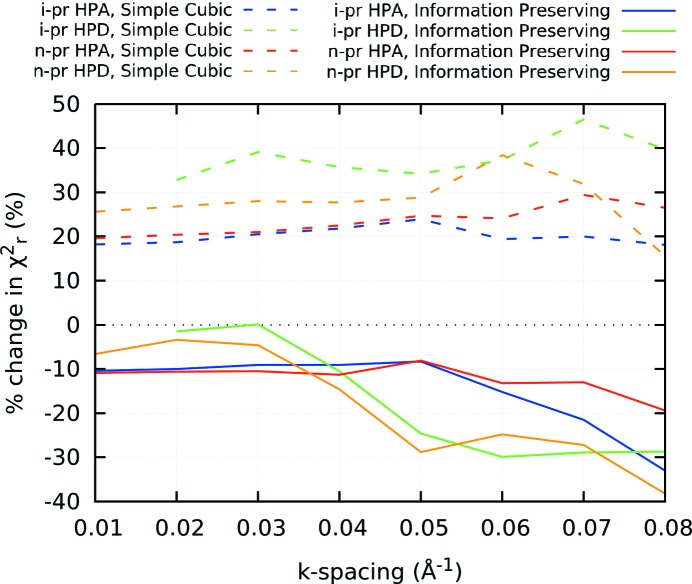
Variations of 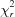 for each data set using the information-preserving algorithm, compared with the respective non-interpolated 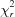. As long as the interpolated grid spacing 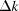 is less than the minimum spacing of the original experimental data set, the approach works very well.

Until now, all interpolation has been performed to a *k*-grid spacing of 0.05 Å^−1^. Fig. 10 ▸ illustrates the percent change in 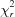 due to interpolation compared with the respective non-interpolated 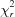 counterpart for each data set for both HPA and HPD data sets.

Fig. 10 ▸ illustrates that the non-corrected cubic interpolated data have increased 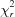 by around 20–40% in all cases. When using the information-preserving uncertainties, this changes to a difference of only approximately 10% for the HPA data, for all interpolation grids with 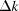 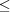 0.05; for 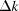 > 0.05, 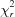 becomes lower due to missed intervals as described above. This effect is seen again with the higher density HPD data with 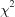 being restored to within 5% for 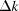 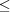 0.03 before dropping rapidly with increasing grid spacing, due to lost intervals; this is an issue for HPD data at a lower *k* spacing than for HPA. Hence the interpolation grid spacing should be chosen carefully for a given data set: as long as the interpolation has a finer grid than the raw data, this approach operates reasonably well to preserve information content.

Hence it is recommended that, should interpolation be required, the use of our information-preservation method will go a reasonable way to preserving the 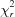 that is representative of the actual (*i.e.* non-interpolated) data.

Whether the raw data are at largely non-uniform spacing, or almost uniform with or without a fine grid, this approach is able to be applied successfully prior to a Fourier transform to position space. It should be emphasized that this method is not appropriate to correct for an oversampled spectrum in *k*-space, as too large a number of intervals will not possess an interpolated point within, and hence the information content is unable to be preserved.

## 10. Effects on refined structure   

To illustrate how the changes in 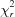 due to interpolation affect structural refinement, Table 9 ▸ shows the bond lengths and angles corresponding to a minimum 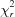 when interpolated data are used, and also when using the error preservation corrected data using the above method.

In all four data sets, using the information-preserving correction on the interpolated data produces structural refinement closer to those found with the non-interpolated data, demonstrating the benefits of local preservation of information content. A particular success is that of the n-pr HPA data, where the corrected interpolation significantly corrected the relatively large errors in all three structural parameters incurred using the initial cubic interpolation.

## 11. Conclusion   

We introduce the idea of avoiding distortions of uncertainties and fits of XAFS by avoiding uniform interpolations in *k*-space from any normal non-uniform experimental data. We provide code and theory for why this should give more insightful results especially for hypothesis testing in comparison of 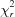 measures or any other goodness-of-fit measure.

Correct propagation of information content gained from high-accuracy experimental techniques throughout the analysis procedure has been demonstrated, and by doing so we have been able to determine new information on the local structures of Ni complexes. The high-accuracy data (HPA) yields nickel bond lengths of 2.017 ± 0.006 Å and 2.022 ∓ 0.006 Å to the nitrogen and oxygen, respectively, and an inter-atomic angle of 85.12 ± 2.0° for the i-pr Ni complex, suggesting a more highly symmetrized tetrahedral arrangement than prior determinations. Contrastingly, this analysis suggests a more skewed square-planar geometry for n-pr Ni complex, with corresponding structural parameters of 2.133 ± 0.004 Å, 1.960 ∓ 0.003 Å and 88.7 ± 3.0° for Ni—N, Ni—O lengths and N—Ni—O angle, respectively. A second method of data collection using a single energy sweep quite like a continuous scan provided similar accuracies and insight and is consistent within defined uncertainty.

We investigate the possibility of deriving uncertainties for a uniform grid and explain some of the difficulties and challenges, including a necessary loss of information and the possibility of missing interpolation intervals. We demonstrate that any such method must yield non-uniform uncertainties even for a uniform grid in *k*-space.

Furthermore we have illustrated the effect interpolation has on the resulting 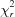, and the magnitude of some consequent errors. We demonstrated that an information-preservation algorithm in our *mu2chi* code, which uses an interval-wise scaling of uncertainties to conserve the local contribution to the final 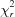, can be quite effective with care on the interpolation spacing relative to the original data.

The methods presented here are important and applicable to all data, whether non-uniform or uniform with a 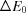 offset, and to any data needing interpolation prior to a Fourier transform. Our recommendations are that using original data uncertainty and avoided interpolation, and ergo fitting in *k*-space, is the most incisive way of hypothesis testing XAFS data. Should transforms be required in a processing environment, it is recommended that a cubic interpolation (not a spline) can preserve the interpolation endpoints and derivatives; and that our presented local information-preserving algorithm provides uncertainties to maintain the potential of hypothesis testing. This fully applies to any continuous scan with or without a 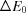 requiring a reinterpolation; or to any continuous scan attempting to have a uniform drive speed in angle or energy; or to any step-wise scans uniform in θ, *E*, *k* or otherwise. 

## Supplementary Material

Code and manual for software. DOI: 10.1107/S1600577518006549/xj5011sup1.zip

## Figures and Tables

**Table 1:** Comparison of XAFS parameters fitted conventionally, *i.e.* without uncertainties and a preliminary refined structure, for Ni—N, Ni—O and N—Ni—O The second and third columns are results using conventional spline-interpolated data (Table 4, Islam *et al.*, 2015 ▸). The same structure is re-fitted with the non-interpolated data in the fourth and fifth columns.

	i-pr Ni (Islam *et al.*, 2015 ▸)	n-pr Ni (Islam *et al.*, 2015 ▸)	i-pr Ni non-interpolated	n-pr Ni non-interpolated
Ni—N(1),(2) (Å)	2.077	2.081	2.077	2.081
Ni—O(1),(2) (Å)	1.976	1.973	1.976	1.973
N—Ni—O (°)	90	90	90	90

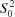	0.999 ± 0.089	0.94 ± 0.12	1.072 ± 0.047	0.791 ± 0.050
α	1.0003 ± 0.004	1.012 ± 0.003	1.0012 ± 0.0069	1.0212 ± 0.0098
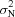 , 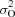 (Å^2^)†	–	–	0.0010‡	0.0010‡
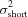 (Å^2^)§	–	–	0.0020‡	0.0020‡
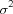 (Å^2^)	0.002 ± 0.0015	0.003 ± 0.002	0.0049 ± 0.0054	0.0071 ± 0.008
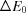 (eV)	0.89 ± 0.87	3.7 ± 1.2	0.95 ± 1.43	5.95 ± 1.78

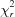	3.21	4.34	5.90	9.69

†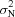 and 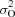 are for the two-legged Ni—N—Ni and Ni—O—Ni paths, set to 0.001 Å^2^.

‡ Fixed to physical value.

§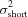 is the thermal broadening parameter for the next shortest 15 photoelectron scattering *FEFF* paths, set to be 0.002 Å^2^.

**Table 2:** Same as Table 1 ▸ with non-interpolated data but using experimental uncertainties in determining the fit

	i-pr Ni	n-pr Ni
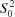	1.094 ± 0.025	0.788 ± 0.028
α	1.0008 ± 0.0032	1.0261 ±0.0062
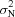 , 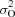 (Å^2^)†	0.0010‡	0.0010‡
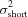 (Å^2^)§	0.0020‡	0.0020‡
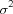 (Å^2^)	0.0048 ± 0.0023	0.0064 ± 0.0044
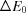 (eV)	0.85 ± 0.72	6.70 ± 1.11
		
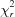	5.905	9.576
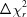	+0.004	−0.117

†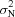 and 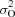 are for the two-legged Ni—N—Ni and Ni—O—Ni paths, set to 0.001 Å^2^.

‡ Fixed to physical value.

§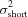 is the thermal broadening parameter for the next shortest 15 photoelectron scattering *FEFF* paths, set to be 0.002 Å^2^.

**Table 3:** Fitted parameters resulting from a two-dimensional (Ni—N, Ni—O) bond length followed by one-dimensional angle refinement

	i-pr Ni	n-pr Ni
Ni—N(1), Ni—N(2) (Å)	2.044 ± 0.006	2.128 ± 0.013
Ni—O(1), Ni—O(2) (Å)	2.007 ∓ 0.006	1.958 ∓ 0.012
N—Ni—O ( 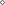 )	85 ± 1	90.24 ± 1.5

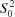	1.07 ± 0.02	0.90 ± 0.03
α	0.9965 ± 0.0029	1.0074 ± 0.0017
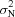 , 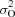 (Å^2^)	0.0010†	0.0010†
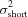 (Å^2^)	0.0020†	0.0020†
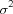 (Å^2^)	0.0047 ± 0.0020	0.0068 ± 0.0034
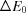 (eV)	−0.72 ± 0.64	4.00†

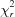	4.971	9.060
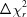	0.934	0.516

† Fixed to physical value.

**Table 4:** Refined structure and cross-fitting of optimized results Refined bond-lengths and angles for the tetrahedral (TD) and square-planar (SQ) theory are determined using i-pr and n-pr data, respectively.

	TD theory	SQ theory
Ni—N (Å)	2.017 ± 0.006	2.133 ± 0.004
Ni—O (Å)	2.022 ∓ 0.006	1.960 ∓ 0.003
N—Ni—O (°)	85.12 ± 2.00	88.7 ± 3.0
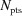 in fit range	79	57

i-pr		
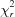	4.809	10.385

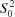	1.079 ± 0.022	1.15†
α	0.9988 ± 0.0028	1.0122 ± 0.0055
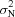 , 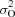 (Å^2^)	0.0010†	0.0010†
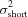 (Å^2^)	0.0020†	0.0020†
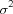 (Å^2^)	0.0050 ± 0.0021	0.0081 ± 0.0034
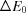 (eV)	−0.42 ± 0.63	4.21 ± 1.08

n-pr		
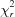	10.504	8.859

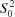	0.756 ± 0.028	0.921 ± 0.031
α	1.0183 ± 0.0063	1.0080 ± 0.0017
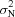 , 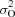 (Å^2^)	0.0010†	0.0010†
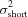 (Å^2^)	0.0020†	0.0020†
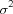 (Å^2^)	0.0024 ± 0.0037	0.0090 ± 0.0034
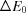 (eV)	3.84 ± 1.16	4.00†

† Fixed to physical value.

**Table 5:** Earlier HPA results (Islam *et al.*, 2015 ▸)

	TD theory	SQ theory
Ni—N (Å)	2.077 (4)	2.081 (4)
Ni—O (Å)	1.976 (4)	1.973 (4)
N—Ni—O (°)	89.2	89.5

i-pr		
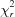	2.94	5.42

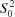	1.02 ± 0.02	1.00 ± 0.21
α	1.0012 ± 0.0033	1.018 ± 0.007
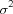 (Å^2^)	0.003 ± 0.002	0.0023 ± 0.002
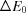 (eV)	0.62 ± 0.28	4.76 ± 1.64

n-pr		
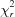	4.72	3.27

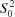	0.93 ± 0.13	0.91 ± 0.02
α	1.008 ± 0.005	1.007 ± 0.003
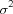 (Å^2^)	0.002 ± 0.002	0.006 ± 0.003
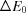 (eV)	1.95 ± 1.26	2.26 ± 1.14

**Table 6:** Three-dimensional refinement of bond length and angle simultaneously using HPD experimental data, analogous to Table 4 ▸

	TD theory	SQ theory
Ni—N (Å)	1.985 ± 0.005	2.115 ± 0.006
Ni—O (Å)	2.055 ∓ 0.005	1.933 ∓ 0.006
N—Ni—O (°)	95.44 ± 2.15	89.49 ± 0.99
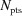 in fit range	162	140

i-pr		
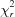	1.894	3.492

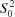	1.009 ± 0.012	1.200 ± 0.020
α	0.9948 ± 0.0022	1.0101 ± 0.0037
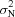 , 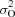 (Å^2^)	0.0010†	0.0010†
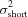 (Å^2^)	0.0020†	0.0020†
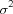 (Å^2^)	0.0075 ± 0.0025	0.03†
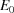 (eV)	−0.52 ± 0.46	1.30 ± 0.02

n-pr		
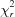	2.046	1.292

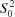	0.95 ± 0.015	1.155 ± 0.014
α	0.9742 ± 0.0029	0.9906 ± 0.0029
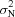 , 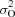 (Å^2^)	0.0010†	0.0010†
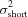 (Å^2^)	0.0020†	0.0020†
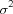 (Å^2^)	0.0067 ± 0.0030	0.0279 ± 0.0047
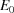 (eV)	−2.85 ± 0.62	−0.65 ± 0.57

† Fixed to physical value.

**Table 7:** Changes in 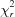 resulting from interpolation of experimental data (interpolation is performed to a *k*-spacing of 0.05 Å^−1^)

		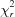	% change
i-pr HPA	Non-interpolated	4.809	
	Cubic	5.944	+23.6%
n-pr HPA	Non-interpolated	7.485	
	Cubic	9.331	+24.7%
i-pr HPD	Non-interpolated	1.894	
	Cubic	2.892	+52.7%
n-pr HPD	Non-interpolated	1.292	
	Cubic	1.664	+28.8%

**Table 8:** 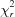
 values for each complex using cubic interpolation of *k*-spacing 0.05 Å^−1^, with information-preserving correction applied Note that the average spacing of the non-interpolated data sets is ∼0.08 Å^−1^ for HPA and ∼0.04 Å^−1^ for HPD.

		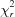	% change	Average β
i-pr HPA	Non-interpolated	4.809		
	Cubic, corrected	4.400	−8.5%	0.54
n-pr HPA	Non-interpolated	7.485		
	Cubic, corrected	6.877	−8.1%	0.75
i-pr HPD	Non-interpolated	1.894		
	Cubic, corrected	1.630	−13.9%	1.38
n-pr HPD	Non-interpolated	1.292		
	Cubic, corrected	0.920	−28.8%	1.42

**Table 9:** Structural refinements using interpolated data The HPA and HPD data sets are interpolated to a spacing of 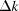 = 0.05 and 0.02 Å^−1^, respectively. The use of the information-preserving correction on the cubic interpolated data goes some way to restore non-interpolated values of structural parameters. That is, it is fairly effective and robust.

	Non-interpolated	Cubic	Cubic, corrected
i-pr (HPA)			
Ni—N (Å)	2.017	2.017	2.019
Ni—O (Å)	2.022	2.018	2.019
N—Ni—O (°)	85.12	86.07	86.03

n-pr (HPA)			
Ni—N (Å)	2.133	2.121	2.133
Ni—O (Å)	1.960	1.939	1.947
N—Ni—O (°)	88.7	84.67	86.45

i-pr (HPD)			
Ni—N (Å)	1.985	1.979	1.980
Ni—O (Å)	2.055	2.062	2.060
N—Ni—O (°)	95.44	95.02	95.03

n-pr (HPD)			
Ni—N (Å)	2.115	2.116	2.114
Ni—O (Å)	1.933	1.931	1.933
N—Ni—O (°)	89.49	89.44	89.49
