# Supplementary material for: Propagation of uncertainty in experiment: structures of Ni (II) coordination complexes
Source: J Synchrotron Radiat. 2018 May 30;25(Pt 4):920–34. doi: 10.1107/S1600577518006549 (PMC6038609; doi:10.1107/S1600577518006549)
Supplement: Supplementary file 1 [file s-25-00920-sup1.zip › mu2chi/mu2chi_manual.pdf]

*** mu2chi installation ***

1. Install *IFEFFIT* or *eFEFFIT*.
2. Replace 'Ifeffit build configuration' section in the attached Makefile with the contents of Config.mak from /usr/local/share/ifeffit/config generated from your prior *IFEFFIT* installation
3. With the Makefile, 'mu2chi.f' and 'ifeffit.inc' in the same directory, execute 'make'. An executable titled 'mu2chi' will be generated

---

*** mu2chi operation ***

1. Prepare experimental input data file. This file should contain 3 columns: Energy, mu/rho, Uncertainty.

2. Configure 'input_params.txt' with desired input parameters. This file should be in the same directory as the mu2chi executable. A list of available options are below. To ensure correct reading of input, enter each keyword on a new line with the desired value separated by a single space, in lowercase. (See example). The only mandatory option is the input data file, which should be specified on the first line. All other options will adopt a default value listed below if failed to be specified.

3. Excute the program by running './mu2chi' from a terminal window.

4. Key details will be output in the terminal window. Besides the primary output data file, a supplementary file 'supp_data.dat' is also produced, containing various arrays from different stages in the program.

5. Use output to fit with model using ifeffit, etc.

*** Summary of Options ***

'inputfile' (mandatory) Sets experimental input data. Relative directories can be specified.

'outputfile'
Specifies primary output.
File extension '.dat' added automatically.
DEFAULT: 'mu2chi_out'

'interpolation':
Set to 'N' for Non-Interpolated output. (recommended)
Set to 'C' for Cubic interpolation.
Set to 'L' for Linear interpolation (not recommended)
DEFAULT: 'N'

'error_preservation':
true/false
Option to use locally preserve information content in interpolated output.
Recommend to set to 'true' (only used for interpolation option 'C')
DEFAULT: true

'display_information_loss':
true/false
Will print out (in terminal) details of intervals that were 'lost' ie: contain no interpolated data point. This may aid in determining appropriate interpolation spacing.
DEFAULT: true

'k-space':	Defines interpolated k-spacing in Angstrom^-1<br>(only used for interpolation options 'C' and 'L')<br>DEFAULT: Determined automatically (please confirm a reasonable value was used by checking terminal output. (See below))
'fit_min'/'fit_max':	k-range that the error preservation optimisation will be performed over. This should be the range that the subsequent fit, eg: in *IFEFFIT,* will ultimately be performed. These values must be set independently again when running such fit.<br>(only used when interpolation set to 'C' AND 'error_preservation' set to 'true')<br>In Angstrom^-1<br>DEFAULT: fit_min=3.0, fit_max=10.0
'e0':	Edge energy in eV for pre-edge and background spline determination<br>DEFAULT: Automatically determined (by turning point of derivative).
'nknots':	Adjusts number of knots used in background spline.<br>Knots are points on the spline where its value and that of the first and second derivatives are continuous. Equally spaced in k-space. [1]<br>Too many knots results in following the spectrum too closely, and hence erasing the information in the XAFS oscillations. (The internal *IFFEFFIT* subroutine used has the ability to override this parameter if deemed unrealistic)<br>DEFAULT: 7
'rbkg':	Parameter used in background spline.<br>Defined as “Upper limit of the low-R region over which the background is to be fit, typically about half the distance of the first shell peak.” [1]<br>DEFAULT: 1.0
'kweight':	Parameter used in background spline.<br>Scales data by k^kweight prior to determining spline.<br>DEFAULT: 2
'pre1'/'pre2':	Sets range in eV relative to edge e0 used to estimate pre-edge.<br>DEFAULT: pre1=-10, pre2=-30
'plots':	true/false<br>Generates plots when set to 'true' (requires GNUPLOT)<br>DEFAULT: true

***** It is strongly recommended that the user visually inspect the output plots generated, to ensure that the pre-edge and background spline appears reasonable.**

[1]. M. Newville *et al.* Phys.Rev. B **47**, 14126 – (1993)

*** Note on Flattening feature ***

This program contains a feature to automatically detect an upward trend in the xafs spectrum. In a fluorescence experiment, the rising trend with energy is due to self-absorption of the fluorescent photon. The program looks the background spline fitted at two data points (50 and 60 points past the edge), and checks for a positive gradient. If detected, the user will be presented with a prompt whether or not to perform a flattening procedure. The user shoud consider if is is appropriate to proceed.

This works by forcing the oscillations in upward trending data post edge to be centred around a flat background. We recognise that this is not a full solution, but it improves the result for limited data sets and in the interim until a comprehensive software package is available to resolve this.

*** Example 1 of input parameter file data ***

```
inputfile data/input.dat
outputfile my_output1
interpolation N
nknots 6
```

*** Example 2 of input parameter file data ***

```
inputfile data/input.dat
outputfile my_output2
interpolation C
error_preservation true
kspace 0.05
fit_min 3.3
fit_max 9.0
e0 8348.52
```

*** Example of experimental input data ***

E	MU	MU_ERR
7918.6	14.3975	0.344
7958.94	13.857	0.128
7998.73	12.4021	0.2331
8039.15	12.8614	0.1579
8078.88	11.5823	0.2661
8078.93	10.7749	0.2019
8119.13	11.2833	0.1043
8159.17	10.8239	0.1782
8199.09	11.4329	0.1137

(only first few rows shown)

*** Example of terminal printout ***

```
Information lost at interval beginning at   8.7325305817873691
Information lost at interval beginning at   9.3088769052201030
Information lost at interval beginning at   9.8564799672638284
Information lost at interval beginning at   12.656760356180220
Information lost at interval beginning at   15.459596502137265
Information lost at interval beginning at   17.530100366470126




***************************************
******* mu2chi PROGRAM COMPLETE *******

--------------------------------------
Cubic Interpolation - with error correction
k-spacing:          0.05
Fit Range:          3.3 - 9.0

Total Points in:      109
Total Points out:     350
Total Intervals lost:  6
--------------------------------------

Output written to "./data6/test.dat"
Additional output written to "supp_data.dat"

pre-edge plotted in "preedge.png"
background spline plotted in "background.png"
final E vs mu data plotted in "final_emu.png"
Chi v k data plotted in "chivk.png"

Have a nice day.
***************************************
```

*** Example of primary output data ***

```
#------------------------
#   k        chi        dchi        e0
  0.50000000E-01   0.50264713      0.24469701E-02    8348.5200
  0.10000000       0.49373289      0.24761132E-02    8348.5200
  0.15000000       0.48481866      0.24905031E-02    8348.5200
  0.20000000       0.47590443      0.24959993E-02    8348.5200
  0.25000000       0.46699020      0.24948565E-02    8348.5200
  0.30000000       0.45807597      0.24881100E-02    8348.5200
  0.35000000       0.44916174      0.24761995E-02    8348.5200
  0.40000000       0.44024750      0.24591783E-02    8348.5200
```

(only first few rows shown)
